# Burst Pressures in Eyes with Clear Corneal Incisions Treated with ReSure Glue

**DOI:** 10.1155/2021/6691489

**Published:** 2021-02-04

**Authors:** Ahmed E. M. Shehata, Siva P. Kambhampati, Jiangxia Wang, Uri S. Soiberman

**Affiliations:** ^1^Cornea Division, Wilmer Eye Institute, Johns Hopkins University School of Medicine, Baltimore, MD, USA; ^2^Center for Nanomedicine, Wilmer Eye Institute, Johns Hopkins University School of Medicine, Baltimore, MD, USA; ^3^Department of Biostatistics, Johns Hopkins University Bloomberg School of Public Health, Baltimore, MD, USA

## Abstract

**Purpose:**

This study aims to measure burst pressures in 3 mm clear corneal incisions sealed with ReSure, a biodegradable hydrogel sealant, and to compare it to traditional 10-0 nylon sutures and unsealed controls.

**Design:**

An ex vivo animal study.

**Methods:**

3 mm clear corneal incisions were performed in rabbit eyes (ex vivo). The burst pressure was determined, and then, the incisions were sealed with either ReSure glue or a single 10-0 nylon suture. Burst pressure measurements were repeated.

**Results:**

Fourteen eyes were included. The median burst pressure in the suture-control group (7 eyes) prior to suture application was 7 mmHg (range: 0–45); the median burst pressure in the 7 glue-controls was 36 mmHg (range: 5–61, *p* = 0.08 for the comparison of the two control groups). The median burst pressure in the glue group was 93 mmHg (range: 39–129, *p* = 0.043 when compared to glue-control). The median burst pressure in the suture group was 158 mmHg (range: 70–180, *p* = 0.018 when compared to suture-control). There was no statistically significant difference in burst pressure values between the glue and suture groups (*p* = 0.08).

**Conclusion:**

In this study, ReSure glue applied to 3 mm clear corneal incisions provided sufficient resistance to elevated intraocular pressure when compared to controls. The results of this study suggest that ReSure glue may be comparable to a single 10-0 nylon suture in resisting fluid egress during the early postoperative period.

## 1. Introduction

ReSure® (Ocular Therapeutix, MA, USA) is a biodegradable polyethylene glycol-based hydrogel designed for use as a corneal incision sealant. Previous studies have demonstrated its efficacy in preventing leakage from clear corneal incisions (CCIs) that are 4.2 mm in length or smaller [1, 2]. However, these investigations, performed in human subjects, were unable to evaluate burst pressures in CCIs sealed with ReSure glue. In this study, we compare burst pressures in 3 mm CCI sealed with either ReSure or a 10-0 nylon suture and compare them to unsealed controls.

## 2. Materials and Methods

Rabbit eyes were purchased from Pel Freeze Biologicals (Rogers, AR, USA). Only mature, mixed gender rabbit eyes were used. The eyes were delivered in Dulbecco's Modified Eagle's medium on ice within 12 hours of enucleation. All experiments were performed no later than 12 hours from delivery.

**Figure 1:**
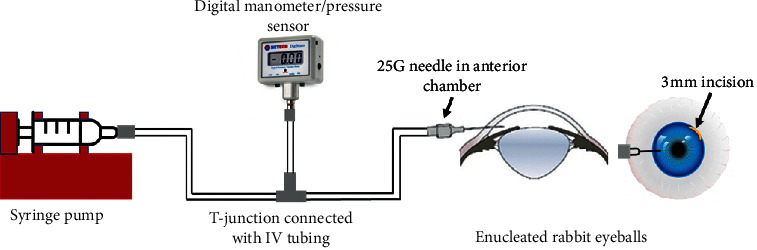
An illustration of the experimental model.

The experiments were performed under a Unitron Z650HR dissecting microscope (Feasterville, PA, USA). Only eyes with clear corneas were used. The anterior chamber was cannulated through the trabecular meshwork with a 30G needle connected to a syringe pump (NE1000 New Era Pump Systems Inc., Farmingdale, NY, USA). The syringe was filled with phosphate buffered solution, and the tubing system was connected to a manometer (Digimano-1000, Netech, Farmingdale, NY, USA). The limbus was marked with a caliper to indicate the borders of a 3 mm incision, and a 3 mm keratome blade (Laseredge, Bausch&Lomb, Rochester, NY, USA) was then used to create the corneal incisions (Figure 1). The incisions were designed to have biplanar architecture.

**Figure 2:**
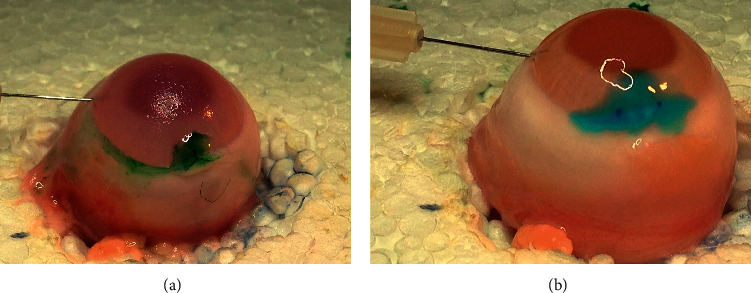
(a) A 10-0 nylon suture was placed on the main corneal wound. Indocyanine green was instilled on the wound to facilitate the identification of fluid egress. (b) Caliper marks are visible in this image. The wound was covered completely with ReSure glue.

A drop of diluted indocyanine green (Akorn Inc, Lake Forest, IL, USA) was placed on the incision to facilitate the detection of fluid egress, while phosphate buffer solution was injected into the eye through the cannula. The pressure at which fluid egress was identified under the microscope, which was confirmed by two investigators (SPK and USS), was defined as the burst pressure. This group of baseline measurements was defined as suture-control for the group of eyes in which the limbal incision was sutured or glue-control for the group of eyes that were later glued. Eyes were consecutively assigned to each group. Eyes with constant unprovoked leakage (i.e., burst pressure 0 mmHg) could not be glued as ReSure glue can only polymerize on dry surfaces. Therefore, these eyes were assigned to the suture group. After this stage was completed, the incision was either sutured with a single 10-0 nylon suture (Figure 2(a); Ethilon CS160-6, Ethicon, Somerville, NJ, USA) or dried with a Weck-Cel sponge (Beaver Visitec, Waltham, MA, USA) and then glued with ReSure glue according to the manufacturer's instructions (Figure 2(b)). The burst pressure was then reassessed. The glue was allowed to polymerize on the wound for one minute before fluid was injected into the chamber to assess burst pressure. The infusion rate was the same in all burst pressure measurements.

### 2.1. Statistical Analysis

Statistical analyses were performed using Stata 13.1 (StatCorp, College Station, TX). The Wilcoxon signed-rank test was used to compare burst pressure values between paired groups (glue vs. glue-controls; suture vs. suture-controls). The Mann–Whitney nonparametric test was used to evaluate the differences between the groups (control groups; glue vs. suture).

## 3. Results

The median burst pressure in the suture-control group prior to glue application was 7 mmHg (*n* = 7, range: 0–45), whereas in the glue-control eyes, it was 36 mmHg (*n* = 7, range: 5–61). The difference between baseline measurements of control groups did not reach statistical significance (*p* = 0.08). After wound enforcement, the median burst pressure in the sutured group was 158 mmHg (*n* = 7, range: 70–180, *p* = 0.018 when compared to suture-control); in the glue group, it was 93 mmHg (*n* = 7, range: 39–129, *p* = 0.043 when compared to glue-control). Comparison of burst pressure measurements between the suture and glue groups revealed no statistically significant difference (*p* = 0.08).

## 4. Discussion

This study demonstrates that ReSure glue applied to 3 mm clear corneal incisions provides ample protection against elevated intraocular pressure at least in the short period of time after its placement. The difference in burst pressure between glued eyes and sutured eyes did not reach statistical significance; however, it is plausible that a tight, well-placed suture will provide better protection against very high intraocular pressure at the cost of inducing a considerable amount of astigmatism. Although rare, sutures may lead to potential complications such as persistent epithelial defects, foreign body sensation, corneal neovascularization, or suture abscesses. Regardless, protection against intraocular pressure higher than 85 mmHg is generally not required from either wound sealing method [3].

A previous study with ReSure glue in patients undergoing cataract surgery with clear corneal incisions of 3.5 mm length or less evaluated leakage in glued incisions compared to sutured incisions and found that the latter had a higher incidence of leaks [1]. The leaking sutured incisions were also shown to require less provocation to induce the fluid egress by applying force to the posterior lip of the incision. The main advantages of that study were its large size (approximately 500 patients), its randomized prospective design, and its duration of follow-up which spanned up to one week postcataract surgery; however, since human patients were enrolled in the study, burst pressures were not assessed. Additionally, in our present study, the suture group did not demonstrate more leakage than the glue group, a finding we attribute to the ex vivo design of the study.

An additional study assessed the use of ReSure glue in endothelial keratoplasty using 4.2 mm clear corneal incisions in 11 eyes [2]. It also demonstrated no leakage at postoperative days 1 and 7. Although this study examined larger corneal cuts, it was also unable to determine burst pressures.

Another retrospective case series compared the use of ocular sealant (ReSure glue) vs. nonsealant in 90 eyes and reported no wound leak in the first postoperative day in both groups. In addition, there was no statistically significant difference between the groups with respect to IOP, clinically noted corneal edema, and foreign body sensation [4]. A recent systematic review by Tan et al. has also emphasized a potential advantage of ocular sealants in comparison to sutures, with the former outperforming the latter in terms of foreign body sensation and surgically induced astigmatism when compared to sutured closure of CCI [5]. In the present study, burst measurements in all groups showed a considerable amount of variability. The variability in burst pressure in the sutured eyes can be explained by suture tension, with tighter sutures having higher burst pressures; however, these sutures also caused more corneal deformation. Variability may also be related to the amount of glue applied to each wound; here, we standardized the approach by utilizing manufacturer's instructions for each application. Some variability can be explained by difference in wound architecture, as corneal incisions were manually created using a 3 mm keratome blade. Additionally, varying degrees of corneal edema in the ex vivo model may have also accounted for this variability in wound applanation; however, the variability of baseline burst pressure between both groups prior to wound enforcement was not statistically significant (*p* = 0.08). Furthermore, the study was designed to allow eyes from each group to act as their own control in order to minimize such difference. Although all wounds were created in a biplanar fashion, even in cataract surgery in vivo, some wounds behave differently than others despite similar architecture. Therefore, these leaking wounds precluded the use ReSure glue, as continuous fluid egress led to glue dilution and prevented proper polymerization.

This study has a few notable limitations: it was performed ex vivo in rabbit eyes rather than in living human eyes; it only provides a snapshot assessment of the burst pressure immediately after the application of the glue or the suture, and it does not assess wound dynamics over time. Another limitation is the small sample size; however, it does contribute data regarding the efficacy of ReSure glue in resisting elevated intraocular pressure and suggests that this glue may be efficacious in sealing CCIs that can be dried completely for a sufficient duration of time.

## 5. Conclusions

The study demonstrates that ReSure glue applied to 3 mm clear corneal incisions provided sufficient resistance to elevated intraocular pressure when compared to controls. The results of this study also suggest that ReSure glue may be comparable to a single 10-0 nylon suture in resisting fluid egress during the early postoperative period.

## Data Availability

The data used to support the findings of this study are available from the corresponding author upon request.
